# Analysis of spatial genetic variation reveals genetic divergence among populations of *Primula veris* associated to contrasting habitats

**DOI:** 10.1038/s41598-017-09154-9

**Published:** 2017-08-18

**Authors:** Pablo Deschepper, Rein Brys, Miguel A. Fortuna, Hans Jacquemyn

**Affiliations:** 1 Division of Plant Ecology and Systematics, Biology Department, University of Leuven, Leuven, Belgium; 2 Research Institute for Forest and Nature, Gaverstraat 4, B-9500 Geraardsbergen, Belgium; 3 Department of Evolutionary Biology and Environmental Studies. University of Zurich, Zurich, Switzerland

## Abstract

Genetic divergence by environment is a process whereby selection causes the formation of gene flow barriers between populations adapting to contrasting environments and is often considered to be the onset of speciation. Nevertheless, the extent to which genetic differentiation by environment on small spatial scales can be detected by means of neutral markers is still subject to debate. Previous research on the perennial herb *Primula veris* has shown that plants from grassland and forest habitats showed pronounced differences in phenology and flower morphology, suggesting limited gene flow between habitats. To test this hypothesis, we sampled 33 populations of *P. veris* consisting of forest and grassland patches and used clustering techniques and network analyses to identify sets of populations that are more connected to each other than to other sets of populations and estimated the timing of divergence. Our results showed that spatial genetic variation had a significantly modular structure and consisted of four well-defined modules that almost perfectly coincided with habitat features. Genetic divergence was estimated to have occurred about 114 generations ago, coinciding with historic major changes in the landscape. Overall, these results illustrate how populations adapting to different environments become structured genetically within landscapes on small spatial scales.

## Introduction

Understanding how the environment shapes species is a central question in evolutionary biology^1–4^. When species are distributed across large areas, geographical isolation can limit gene flow and cause strong genetic differentiation, ultimately leading to ecotype and even species formation. This process can take place either along an ecological gradient or across isolated, well-defined habitat entities^1, 5, 6^. Strong genetic differentiation can also occur at much smaller local scales as a result of contrasting environmental features that affect gene flow and population genetic structure^7–10^. It has for instance been observed that recently diverged sister species originate from habitat-specific adaptations following colonization events in new environments^11–14^. Similarly, landscape heterogeneity can lead to formation of locally adapted populations with reduced gene flow between habitats.

Several reproductive barriers that prevent gene flow can arise when a species occupies contrasting environments within the same geographical region, which in turn can elicit genetic structuring^15, 16^. Prezygotic barriers such as differences in flowering time significantly reduce gene transfer between neighboring populations growing under contrasting environmental conditions or in different habitats^17–21^. Additionally, differences in pollinator communities between habitats can shape floral morphology and therefore significantly impact on gene flow by pollen transfer^22–25^. Different habitats also demand different adaptations in a way that the right alleles are required to thrive in a specific environment^26^. This restricts certain genotypes to settle or flourish in a given environment characterized by specific soil characteristics, predators, or light availability^4, 21, 27, 28^. Selection against immigrants due to maladaptation hampers integration of genes of less fit genotypes into the native genotype and can for this reason be considered as an additional legitimate barrier against gene flow and thus ultimately a driver for genetic divergence among populations^29–32^.

However, how these aspects affect gene flow at neutral loci between diverged populations has only been studied recently^3, 19–21^. Because ecological divergent selection can give rise to barriers to gene flow^33^, which in turn may reduce the homogenizing effect of gene flow among habitats, this can cause genetic divergence at neutral loci between habitats due to genetic drift^10, 33^. Demonstrating isolation by adaptation with neutral markers is not always possible since this process largely depends on the stochastic nature of genetic drift or the presence of weakly linked genomic regions under divergent selection^33^. Nonetheless, several studies have recently used neutral markers to assess population genetic structure and its relation to natural selection^10, 32, 34, 35^. Direct comparison between neutral microsatellite markers and a genetic marker directly influenced by natural selection has shown that microsatellites are better suited to determine population genetic structure as a consequence of neutral processes such as gene flow^36^. Furthermore, a simulation study showed that ecological differences can cause a reduced gene flow at neutral markers resulting in genetic differentiation among populations^37^.

Forests and grasslands represent two contrasting habitats that largely differ in several environmental conditions, such as light availability, humidity and temperature, which in turn may select for different traits and result in ecological divergence. Recent research on the distylous *Primula veris* has shown clear evidence for habitat-specific differentiation in phenology and flower characteristics between neighboring populations growing in forests and grasslands^38^. Due to increasing shade during the growing season, plants in the forest habitat flower about three weeks earlier compared to neighboring populations growing in open grasslands, restricting the chances of gene flow between populations of both habitats^38^. The size of the flowers and positioning of the anthers and stigma also differed significantly between grassland and forest plants. In particular, the L-morph flower of forest plants showed strong deviation from anther-stigma separation^38^. Deviations from reciprocal placement of sexual organs can hamper pollen deposition on compatible stigmas^25^ and therefore restrict pollen flow between grassland and forest populations. Due to these differences, we hypothesized that restricted gene flow between forest and grassland populations has created genetically structured groups within a mosaic landscape consisting of both grassland and forest habitats and therefore may present the very first signs of ecological speciation.

To test this hypothesis, we investigated patterns of genetic variation and structure in a large set of populations of *P. veris* using 12 polymorphic microsatellite loci. We used Bayesian clustering techniques and a landscape genetic analysis based on network theory to test whether grassland and forest populations can be divided into clusters that act as independent evolutionary units in the landscape and to see whether these clusters can be brought back to specific characteristics of the habitats they were sampled from. Additionally, coalescent-based approximate Bayesian computation (ABC) was used to make inferences about population history and to estimate the timing of divergence between populations growing in grassland and forest populations.

## Results

### Analysis of genetic diversity

Population sizes ranged from 60 to approximately 12 000 flowering individuals (Table 1). There was no significant (*P* > 0.05) difference in population size between grassland (average size: 345 ± 26.35) and forest populations (207 ± 167.81) after omitting one outlier. In total, 119 different alleles were found across 12 microsatellite marker loci in 792 sampled plants. The number of alleles per locus ranged from four to 23 (mean: 9.92). Measures concerning genetic diversity are summarized in Table 1 with mean values given for both habitats. The number of alleles (*P* = 0.09, *t* = 1.73, *df* = 31) and allelic richness (*P* = 0.17, *t* = 1.41, *df* = 31) were not significantly different between both habitats. Expected (*H*_*e*_) and observed (*H*_*o*_) heterozygosity ranged from 0.325 to 0.606 and from 0.385 to 0.589 in grassland and forest populations respectively and did not differ significantly between habitats (mean *H*_*e*_ grassland: 0.535 and mean *H*_*e*_ forest: 0.529, *P* = 0.67, *t* = 0.43, *df* = 31; mean *H*_*o*_ grassland: 0.475 and mean *H*_*o*_ forest: 0.466, *P* = 0.70, *t = *0.38, *df* = 31). The mean values for *F*_*is*_ were positive for both habitats (mean grassland: 0.136; mean forest: 0.119), indicating slight inbreeding. The inbreeding coefficient did not differ significantly between habitats (*P* = 0.60, *t* = 0.63, *df* = 31). All but two loci showed significant heterozygote deficiency across all populations. Linkage disequilibrium (LD) was found in 7 out of 66 possible locus pairs following Bonferroni correction. Significant linkage for at least one locus pair was present in three grassland and three forest populations (populations 1, 2, 11, 19, 27 and 30).

**Table 1:** Genetic diversity measures for all sampled *P. veris* populations among the two habitats.

Habitat	ID	Size	N	A	A_r_	H_o_	H_e_	F_is_
Grassland	1	210	24	4.7	2.80	0.538	0.590	0.083
Grassland	2	490	24	4.3	2.46	0.454	0.492	0.101
Grassland	3	85	24	4.7	2.68	0.494	0.532	0.092
Grassland	4	100	24	4.6	2.63	0.525	0.543	0.053
Grassland	5	100	24	5.3	2.86	0.576	0.574	0.007
Grassland	6	100	24	5.2	2.71	0.486	0.541	0.132
Grassland	7	70	24	5.6	2.88	0.606	0.585	0.031
Grassland	8	200	24	4.3	2.59	0.325	0.500	0.414
Grassland	9	750	24	4.9	2.79	0.521	0.569	0.107
Grassland	10	550	24	4.5	2.46	0.444	0.467	0.037
Grassland	11	1250	24	5.2	2.69	0.434	0.518	0.170
Grassland	12	12000	24	5.0	2.60	0.453	0.512	0.133
Grassland	13	220	24	4.5	2.54	0.440	0.481	0.195
Grassland	14	420	24	4.9	2.83	0.494	0.576	0.176
Grassland	15	160	24	5.3	2.82	0.439	0.555	0.269
Grassland	16	90	24	5.0	2.76	0.538	0.558	0.050
Grassland	17	860	24	4.6	2.59	0.388	0.526	0.162
Grassland	18	210	24	4.8	2.61	0.391	0.519	0.291
Grassland habitat mean				4.9	2.68	0.475	0.535	0.136
Forest	19	320	24	4.4	2.80	0.548	0.615	0.091
Forest	20	400	24	4.0	2.75	0.537	0.623	0.104
Forest	21	110	24	5.1	2.96	0.589	0.633	0.027
Forest	22	210	24	5.2	2.44	0.410	0.463	0.186
Forest	23	190	20	3.9	2.51	0.429	0.496	0.160
Forest	24	90	20	4.1	2.58	0.385	0.478	0.170
Forest	25	60	24	5.0	2.74	0.420	0.546	0.225
Forest	26	90	24	4.5	2.66	0.439	0.549	0.190
Forest	27	70	24	4.4	2.50	0.471	0.497	0.047
Forest	28	210	23	4.4	2.55	0.451	0.496	0.124
Forest	29	380	19	4.8	2.58	0.450	0.520	0.117
Forest	30	60	24	4.3	2.48	0.462	0.492	0.055
Forest	31	680	24	5.5	2.67	0.477	0.538	0.105
Forest	32	160	24	4.7	2.42	0.452	0.472	0.087
Forest	33	75	24	4.8	2.58	0.474	0.510	0.100
Forest habitat mean				4.6	2.61	0.466	0.529	0.119

Size, number of flowering individuals; N, number of sampled individuals; A, number of alleles per locus; A_*r*_, allelic richness; H_*o*_ *, observed heterozygosity, H*_*e*_, expected heterozygosity; F_*is*_, inbreeding coefficient. The mean value of every genetic measure is given for each habitat.

### Genetic structure and isolation by distance

**Figure 1:**
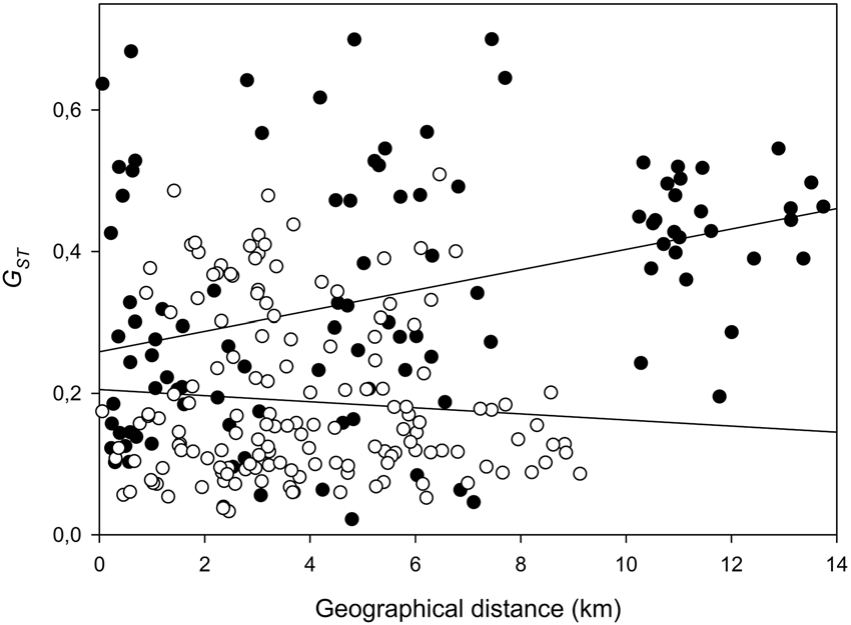
Relationship between geographic distance and *G*_*ST*_ for grassland (white dots) and forest (black dots) populations.

The AMOVA analysis revealed that most of the genetic variation was found between individuals within populations (81%), whereas 14% of variation was due to differences between populations and 4% due to differences between habitats. All values were highly significant with *P* < 0.001 (Table 2). Overall genetic differentiation was moderate (*F*_*ST*_ = 0.069, *G*_*ST*_ = 0.263, Jost’s *D* = 0.204) (Table 3). The ANOVA-like Mantel test indicated that genetic differentiation was significantly larger between habitats than within habitats for every parameter of genetic differentiation (*P* < 0.01) (Table 3). Inspecting the values of the genetic differentiation measures within and between habitats, this result was most likely caused by the higher gene flow between grassland populations (Table 3). Indeed, genetic differentiation was significantly higher between forest populations than between populations of grassland habitats (*F*_*ST*_ = 0.087 and *F*_*ST*_ = 0.052 respectively with *P* < 0.001). Additionally, traditional Mantel tests showed a significant positive relationship between genetic and geographic distance for forest populations (*R* ^2^ = 0.126; *P* < 0.01), whereas no such relationship was detected for grassland populations (*R* ^2^ = 0.008; *P* = 0.225) (Fig. 1).

**Table 2:** Hierarchical analysis of molecular variance based on 12 microsatellite loci and 33 populations.

Source of variation	d.f.	Sum of Squares	Phi-statistics	% of total variance	*P*-value
Between groups	1	244.111	0.043	4.30	< 0.01
Among populations within groups	31	1523.110	0.150	14.35	< 0.01
Among individuals within populations	759	7127.583	0.186	81.35	< 0.01
Total	791	8894.804

**Table 3:** Different parameters for pairwise genetic differentiation given for the two habitats.

	all populations	within grassland habitat	within forest habitat	between habitats
*F*_*ST*_	0.069	0.052	0.087	0.084
*G*_*ST*_	0.263	0.189	0.337	0.326
*D*	0.204	0.125	0.242	0.234

Values for all genetic differentiation parameters between populations of different habitats were significantly different from values between populations of the same habitat as reported in the ANOVA-like mantel test (P < 0.01). Genetic differentiation was also significantly greater amonulations within the forest habitat than between grassland populations for all parameters (P < 0.001).

**Figure 2:**
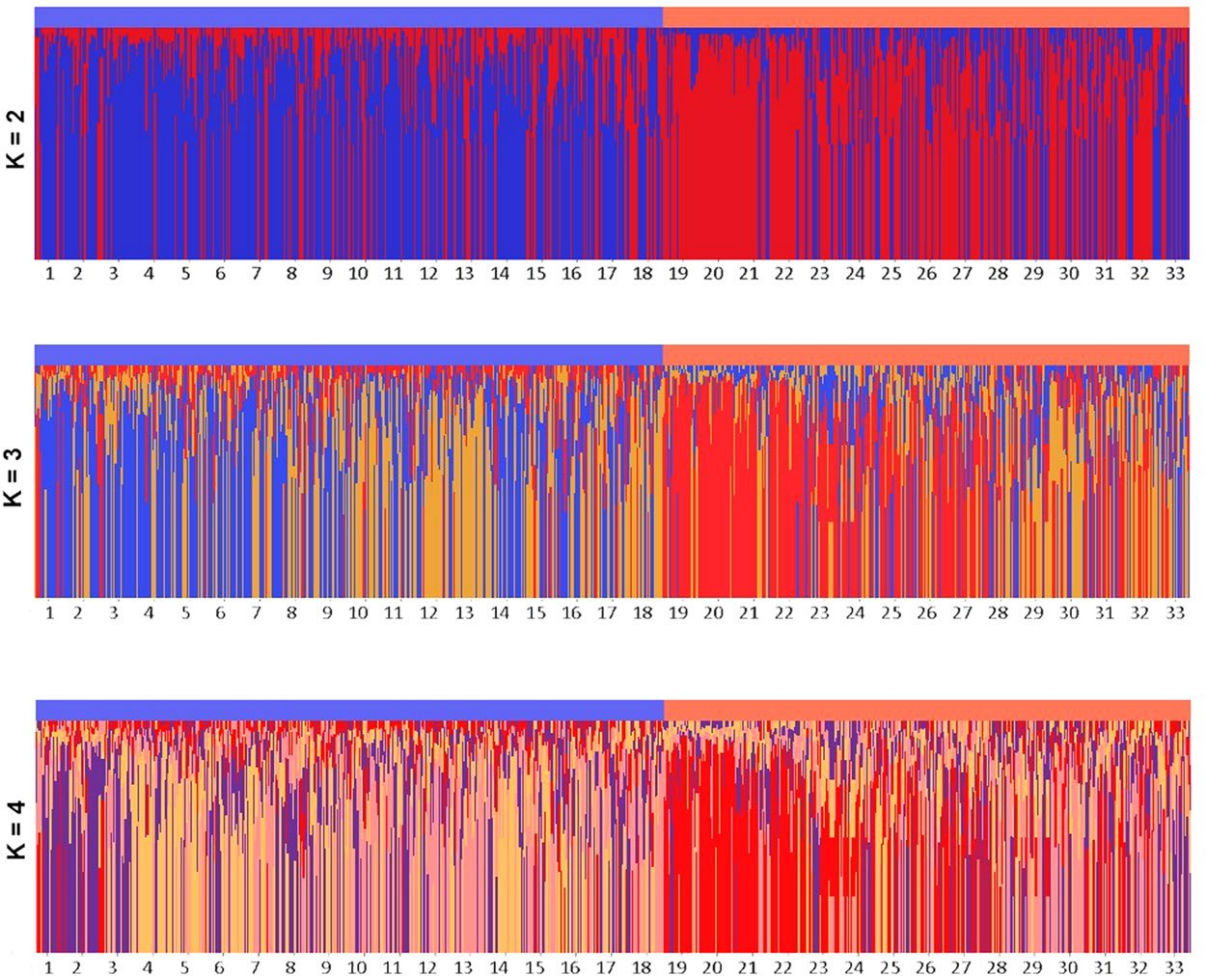
Bayesian cluster analysis (from K = 2 to K=4) for all *P. veris* populations with a color indication for the respective habitat for each of the 33 populations visualized with Structure Plot shiny web application^96^ (blue for the grassland habitat and red for the forest habitat).

Bayesian clustering (Fig. 2) revealed two distinct genetic clusters inferred by Evanno’s ∆*K* ^39^. The inferred clusters strongly corresponded with the habitats from which individuals were sampled, confirming differentiation by environment (∆*K* = 140.8 for *K* = 2, second largest *∆K* = 11.5 for *K* = 3, third largest *∆K = *5.9 for *K* = 4). The average *Q* values for grassland populations were 0.705 and 0.295 for the red and blue cluster respectively, and 0.321 and 0.679 for the forest populations. The value for *LnP(D)* reached a plateau at *K* = 4 and corresponding cluster plots are shown in Fig. 2. We did not find a different optimal number of clusters when populations that showed linkage at one or more loci were omitted from the clustering analysis (∆*K* = 26.2 for *K* = 2, second largest *∆K* = 10.1 for *K* = 4, third largest *∆K = *5.8 for *K* = 3).

**Figure 3:**
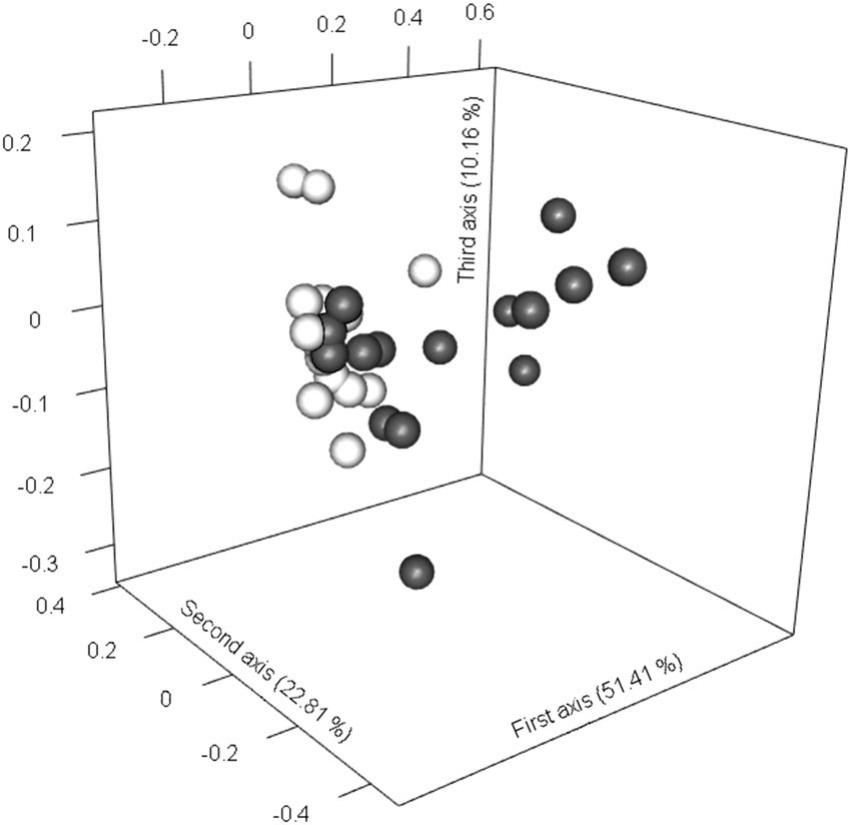
PCoA of the 33 *P. veris* populations with grassland and forest populations indicated in light grey and black respectively. The percentage of explained variation by each axis is shown between the brackets.

Clustering of the populations belonging to the two habitats was also visually confirmed by the PCoA analysis (Fig. 3). Furthermore, distances between forest populations in the PCoA were larger than distances between grassland populations, supporting our previous finding that forest populations were less closely related to each other than grassland populations.

### Networks of spatial genetic differentiation and identification of modules

**Figure 4:**
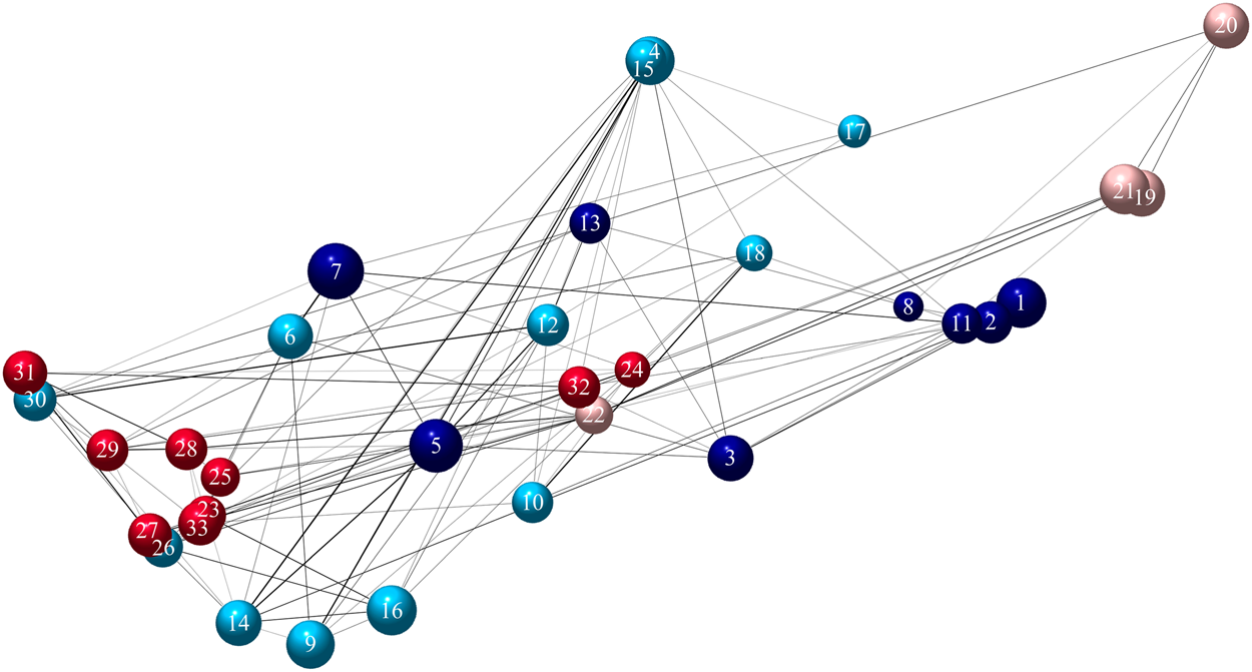
Spatial network of genetic variation with four detected modules (different colors). Links represent significant genetic similarity with line thickness positively correlated to the level of similarity. Node size shows population heterozygosity and node position reflects the geographic position. Population 1 to 18 and 19 to 33 are grassland and forest populations respectively.

The network of spatial genetic variation of *P. veris* populations (Fig. 4) showed 33 nodes connected with 107 links. 43% of the links were established between grassland populations (average number of links per population: 2.56) and 31% between forest populations (average: 2.20). 26% of the links were established between nodes of a different habitat. Overall network connectance (number of remaining links over all possible links) was 0.203. Network connectance was markedly lower between (0.104) than within habitats (0.301 for the grassland habitat and 0.314 for the forest habitat). The overall network showed a significant modular structure (modularity *M* = 0.32; *P* = 0.013). Four different modules were detected (Fig. 4). Almost all the populations within one module corresponded to one habitat type. Only one module contained a mixture of grassland and forest populations (light blue module) with two out of 12 populations belonging to the forest instead of the grassland habitat. The two forest modules (red and light red) have their core on separate sides of the study area, suggesting stronger isolation by distance than the grassland modules.

### Time of divergence and historical gene flow

Results from ABC showed that grassland and forest populations diverged ca. 114 generations ago (95% CI: 126 y - 1030 y). This is 228 y before present assuming a generation time of 2 years. Model checking indicated that summary statistics from the posterior predictive distribution of the simulated datasets correctly estimated parameters. More specifically, none of the summary statistics of the 1% closest simulated datasets deviated significantly from those of the observed dataset (*P* = 0.288–0.925). The MIGRATE-N analysis showed that the mutation-scaled immigration rate *M* was highly directional with migration occurring mainly from the grassland into the forest habitat (*M* = 1043) and far less from the forest into the grassland habitat (*M* = 278).

## Discussion

Identifying the factors that promote genetic differentiation is of interest for understanding the processes initiating the early stages of speciation. Gene flow among populations inhabiting different environments can be reduced by geographical distance or by divergent selection resulting from local adaptation^10, 27, 35, 40, 41^. However, disentangling the relative role of spatial and environmental factors in shaping patterns of population differentiation is not straightforward as both are likely to be intertwined. Several studies have addressed genetic divergence that is attributable to environmental adaptation to the environment^10, 19, 35, 41, 42^. Here, we used microsatellite markers to test the hypothesis that differences in phenology, flower morphology and potentially other adaptions to contrasting habitats (e.g. shade and drought tolerance) between grassland and forest populations of the perennial herb *P. veris* translated into genetic structuring and the occurrence of well-defined genetic entities in the landscape.

Genetic differentiation across all populations was moderate (*F*_*ST*_ = 0.069), with forest populations showing the largest differentiation. The analysis of molecular variance showed that 4% of the total genetic variation could be attributed to differences between habitats, indicating that environmental factors significantly contributed to partitioning of genetic variation and that gene flow seems to be restricted even if populations are located close to each other. In the related *Primula merrilliana*, Shao *et al*.^43^ documented a much higher value of variation that could be attributed to different habitats (13.30%). In this study, however, both contrasting habitats (foothill versus mountain habitat) were clearly spatially separated with extreme differences in growing conditions, both of which can be expected to have a considerable impact on genetic variation. On the other hand, Jacquemyn *et al*.^44^ found lower amounts of variation (<4%) that were associated with patch age in the related forest herb *Primula elatior*. A very similar study on the epiphytic orchid *Fumellea rossi* that grew in different forest types reported a value of 2.1% for the part of the total genetic variation that could be ascribed to habitat ^40^. A comparable value of 2.3% was reported by Andrew *et al*.^35^ in the sunflower *Helianthus petiolaris* occurring in dune and non-dune populations, which they denoted as different ecotypes.

Our results further showed a higher genetic differentiation between forest populations than between grassland populations, suggesting that grassland populations are stronger connected to each other than forest populations. Indeed, isolation by distance is causing partitioning of genetic variation in the forest habitat and not in grasslands probably because of the lack of connecting suitable habitat patches in the landscape. This result is not surprising given that gene flow by seeds is restricted in *P. veris* ^45, 46^ and that most gene flow therefore is the result of pollen flow. Within the study area, populations of *P. veris* can often be found along grassy road verges, which provide corridors for gene flow and essentially connect different grassland populations. Open old growth forests, on the other hand, are not as widely or evenly distributed across the study area and are often surrounded by denser younger forest and separated by agricultural fields or intensively managed grasslands. Moreover, *P*. *veris* shows reduced performance under shade and is therefore not likely to be present in forests with a dense shrub layer^46^, further restricting the presence of *P. veris* to very specific forest conditions. In our case, forest populations clustered within three large forest patches embedded within agricultural fields, which may to some extent have contributed to the isolation-by-distance pattern observed for forest populations. As a result, pollinators have to fly large distances to spread genes from one forest population patch to another. Additionally, it has been observed that bumblebees, the main pollinators of *P. veris* in forests, tend to fly over forests rather than fly through them to be cost efficient^47^, further restricting pollen flow among forest patches.

Differentiation between habitats was considerable (*F*_ST_ = 0.084) and was visualized by a Bayesian cluster analysis and a PCoA, which revealed two distinct groups of populations largely overlapping with the two *P*. *veris* habitats, indicating a strong concordance between the type of habitat and the partitioning of gene diversity in the landscape. This result is similar with findings of Mallet *et al*.^40^, where the number of clusters equaled the number of different habitats. The network analysis of spatial genetic variation largely confirmed these results and showed that genetic variation was spatially distributed across four modules that almost entirely overlapped with populations belonging to the grassland or the forest habitat. Populations within habitats were also more connected to each other than to populations of the other habitat, suggesting that gene flow between forest and grassland populations was restricted. Similar results were shown by Lowry *et al*.^15^, who showed significant genetic clustering between coastal and inland races of *M. guttatus*. However, in contrast with this study, the forest and grassland populations investigated here occurred interspersed within the landscape. The detection of four different evolutionary units by our module finding algorithm therefore also suggests exploration for clustering at a deeper level than the cluster analysis implemented in STRUCTURE^48^.

The presence of significant modularity points to the existence of independent evolutionary units that may form the basis for further adaptation and ultimately ecotype formation. Several adaptations to specific environmental features linked to the habitats in which *P. veris* grows can be expected. First, the observed differences in flowering time may limit the chances for gene flow between forest and grassland populations and can cause genetic differentiation^17, 20, 27^. Differences in flowering time are most likely an adaptation to different light conditions encountered in forest and grassland habitats^49–51^. Previous research has shown that the peak in the light saturated rate of CO_2_ assimilation (Amax) in *P. veris* occurs at the beginning of April, when plants start flowering, and declined during the growing season^52^. *Primula veris* thus shows an increased carbon gain early in spring and exploits the high spring light phase before expansion of the vegetation canopy. This high light phase largely coincides with flower and fruit production, and a substantial carbon gain in spring could guarantee the initiation of flower primordia and/or increase the proportion of dormant flower buds developing^52^. Flowering earlier in the year in forest habitats, before canopy closure, can therefore be considered as a reproductive strategy of *P. veris* to escape competition for light.

Field observations have also indicated that pollinator communities differ between grassland and forest populations, with little overlap between them^53^. Whereas pollinator communities in grasslands are dominated by the hairy-footed flower bee *Anthophora plumipes*, this species was absent in forest populations. Here, bumblebees (*Bombus terrestris*, *B. lapidarius*) and several *Lasioglossum* species were the most frequent insects visiting *P. veris* flowers. Separation of flowering in time and pronounced differences in pollinator communities therefore most likely act as reproductive barriers that limit gene flow between habitats^21, 27, 54^.

Furthermore, successful establishment after immigration to the opposing habitat could be hampered by immigrant unviability because of maladaptation^19, 35, 55^. Grasslands and forest are strongly different habitats, demanding different sets of adaptations which can directly compromise the ability of a plant to germinate, grow or reproduce in a non-native environment^26, 55, 56^. For example, previous research has shown that compared to the related *Primula elatior* and *P. vulgaris*, *P. veris* is least well-adapted to survive moderate shade, as a consequence of its relatively low quantum efficiency, high light saturation point and high dark respiration^52^. The pronounced differences in specific leaf area and stomatal density point to habitat-specific adaptations to light conditions^57^ or water status^58^, which may restrict establishment of grassland plants in forest habitats and vice versa.

The time of divergence between grassland and forest populations was estimated to occur at the end of the 18^th^ century. This period coincides with large alterations of the landscape^59, 60^. In 1775, the date of first map of the study area, the entire area was widely covered with calcareous grasslands and old-growth forest was limited. These grasslands are known for their high diversity in flowering plants^61^ and attract a wider variety of insects because they can supply copious nectar for pollinators^62^. It is therefore reasonable to assume that pollinator diversity was highest in calcareous grasslands and that gene flow occurred predominantly from grassland to forest. This is in line with our results that showed that historical migration rates were about 4 times larger from grassland to forest populations than from forest populations to grassland populations. However, in the 18^th^ and especially the 19^th^ century, many calcareous grasslands were abandoned^60^ or planted with high-productivity forests that mainly consisted of *Pinus nigra*. These secondary forests are unsuitable to support *P. veris* populations and this may have initiated increased isolation between forest and grassland populations. Assuming that, due to the increased isolation, less pollen was dispersed from grasslands into forest populations, forest and grassland populations gradually started to diverge and phenotypic and genotypic differences between forest and grassland populations started to increase.

To conclude, our results demonstrated that populations of *P. veris* formed clear genetic entities in the landscape that were related to the habitat from which they were sampled. Historic changes in landscape configuration and spatial isolation and the associated changes in gene flow probably have gradually induced phenotypic and genotypic differences between plants from grassland and forest populations. Future research is needed to examine whether the observed phenotypical differences, such as specific leaf area or stomatal density, are the result of plasticity or are in fact evolutionary adaptations^63^ and to identify genomic regions that are subject to adaptation.

## Materials and Methods

### Species

*Primula veris* L. (cowslip) is a herbaceous, spring flowering perennial plant species that usually grows in calcareous grasslands, but that can also be found in old growth forest and hedgerows^46^. This rosette forming hemicryptophyte can be found throughout most of temperate Europe and the British Isles until the western Russian border. In early spring, *P. veris* forms a rosette of leaves and produces flowers that grow in umbels. Since *P. veris* prefers a warm microclimate, it has a preference for sunny slopes and open forest patches. Selfing is prevented by a diallelic self-incompatibility system and heterostyly with two reciprocal flower morphs (pin and thrum)^38, 64^. Pollen flow is mainly accomplished by early Hymenoptera (mostly bumble bees and bees) that are able to reach the nectar^46^. Seed dispersal is restricted to only a few meters from the maternal plant since there is no mechanism for long-distance seed dispersal^46^.

Previous research has shown some remarkable differences between forest and grassland plants^38^. Cowslip flowers about three weeks earlier in forest habitats than in grasslands^46^, possibly as a way to take advantage from the higher light availability in spring before canopy closure^49^. Additionally, *P. veris* shows habitat specific variation in flower morphology, with forest populations generally producing larger flowers that show strong deviations in stigma-anther separation, particularly in the L-morph^38^. This deviation is mainly driven by variation in stigma height, resulting in high and asymmetric reciprocity indices and the occurrence of several short-styled homostylous plants^38^. In contrast, flowers of grassland plants show clear distyly with low and symmetric reciprocity indices at both the lower and upper level^38^. Analysis of vegetative traits further shows that plants in grasslands produce more, but shorter flowering stalks. Interestingly, grassland plants have a lower specific leaf area and a higher stomatal density than forest plants (Deschepper, unpublished data), also suggesting habitat specific adaptations to cope with the different environmental conditions imposed by forest and grassland habitats^58, 65^.

### Study area and sampling

**Figure 5:**
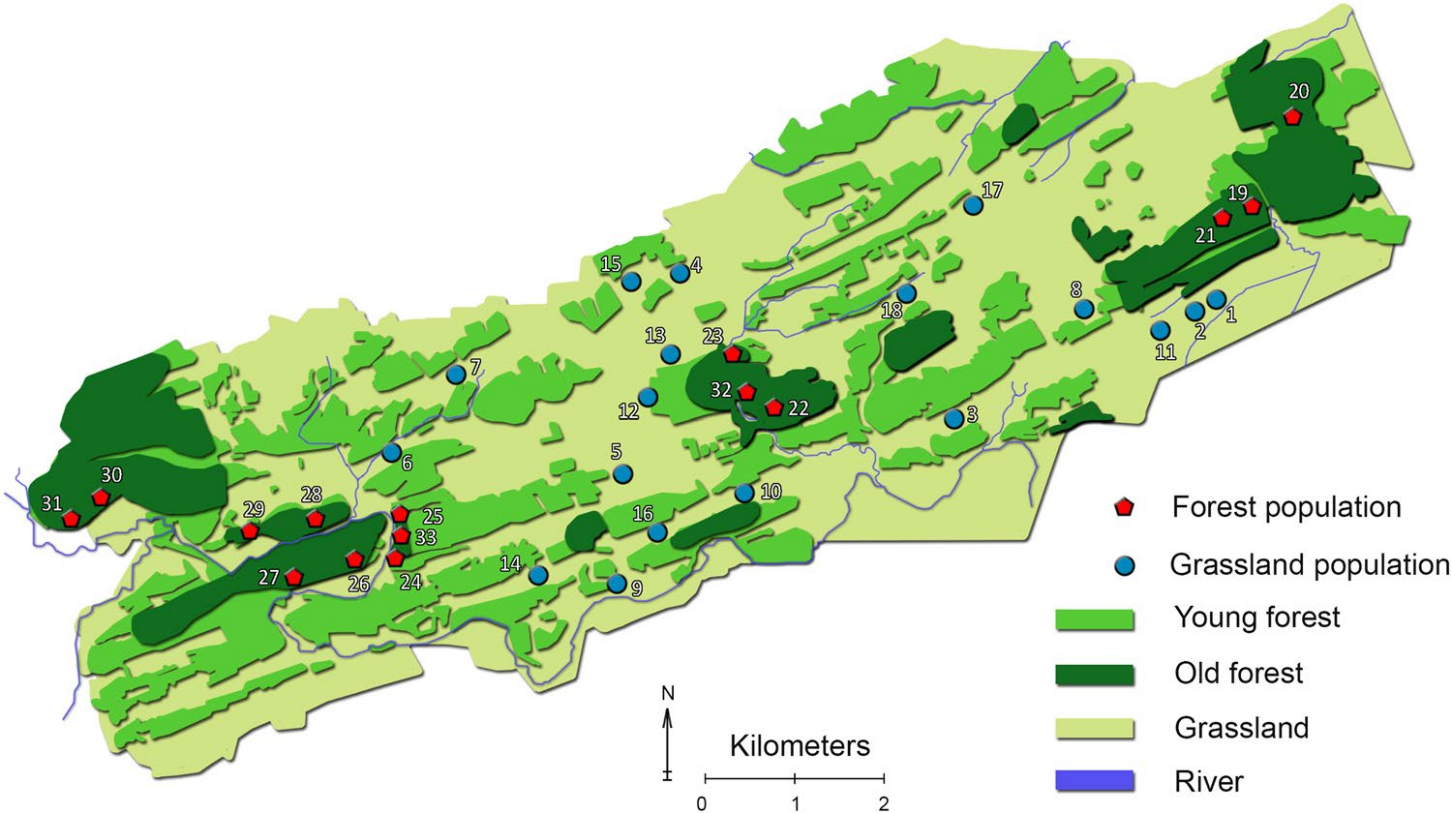
Map of the study area with forest populations indicated by red pentagons and grassland populations by blue circles. Old forest are forests that were present at least from 1775 until today. Map modified with QGIS (www.qgis.org) from OpenStreetMap.org. OpenStreetMap is made available under the Open Database License: http://opendatacommons.org/licenses/odbl/1.0/. Any rights in individual contents of the database are licensed under the Database Contents License: http://opendatacommons.org/licenses/dbcl/1.0/.

The study area is located in the river valley of the Viroin River in the south of Belgium, in the Namur province located in the Calestienne region. The study area consists of a mosaic landscape of forests, calcareous grasslands and agricultural fields and covers a total surface area of 40 km^2^ (Fig. 5). Topographic altitudes within the study area range from 170 m on foothills to 230 m on hilltop locations. Mean annual temperature is 9.8 °C and the average annual precipitation is 780 mm (Royal Meteorological Institute Belgium). More detailed information on the history of the region can be found in Adriaens *et al*.^59^.

In spring 2015, a total of 33 populations on the northern side of the Viroin River were sampled (Fig. 5). Because it is crucial to separate ecological from spatial factors in determining genetic differentiation^10^, populations were sampled for each habitat as uniformly as possible over the whole study area. The sampled populations showed a patchy distribution separated by (intensely) managed farmlands throughout the region (Fig. 5). Forest populations (*n* = 15) were located in open, old growth forests, whereas the grassland populations (*n* = 18) were selected in species-rich calcareous grasslands or grassy road edges. Grazing was common practice in the management of calcareous grasslands in the past and is still applied today^60^. Patches containing *P. veris* populations ranged from approximately 40 m^2^ in size for roadside populations up to several hundreds of square meters in grassland populations. For each population, population size was assessed by counting the total number of flowering plants. Additionally, a large healthy leaf was taken from 24 plants per population and was immediately stored within silica gel until further analyses in the lab.

### DNA extraction and genotyping

20 mg of dry leaf material was weighted and homogenized with the use of zirconia beads and a FastPrep-24 Instrument (MP Biomedicals, USA), after which DNA was isolated using the NucleoSpin® Plant II DNA extraction kit (Macherey Nagel, Germany). DNA quality and concentration were evaluated using a NanoDrop ND-2000 spectrophotometer (Thermo Scientific, Wilmington, DE, USA). For genotyping, 12 primer pairs were used (developed by Bickler *et al*.^66^ and Seino *et al*.^67^ in 3 multiplex reactions for each locus containing 1 µl DNA sample, 5 µl of Qiagen Multiplex PCR Master Mix, 2 µl of one of the multiplexed primer combinations (1 µM for both the forward and reverse primer) and 2 µl of RNAse-free water. Forward primers were fluorescently labeled with one of the four available colours (FAM, VIC, PET and NED). A 2720 Thermal Cycler (Applied Biosystems, CA, USA) was used for the polymerase chain reaction with the following program: an initial denaturation at 94 °C for 3 min, then 10 cycles of 30 s at 94 °C, 60 s at 57 °C and 30 s at 72 °C. This was followed by a further 28 cycles of 94 °C for 30 s, 55 °C for 60 s and 72 °C for 30 s, finishing with a final extension phase at 72 °C for 7 min. After the fragment elongation, capillary electrophoresis was performed using an ABI3130 instrument (Applied Biosystems, USA) using a GeneScan 500 LIZ (Life Technologies, USA) size standard. Microsatellite peaks were visualized and scored using GeneMapper® Software v4.0 (Applied Biosystems).

### Analysis of genetic diversity

Micro-Checker was used to check for null-alleles, stutter and large allele dropout ^68^. We used R packages ‘hierfstat’^69^ and ‘adegenet’ ^70^ in R 3.3.0^71^ to estimate the average number of alleles per locus (*A*), allelic richness (*A*_*r*_), observed (*H*_*o*_), expected heterozygosity (*H*_*e*_) and the inbreeding coefficient (*F*_*is*_) for each population. Linkage disequilibrium was checked with GENEPOP 4.0^72^. Conformance to Hardy–Weinberg equilibrium was determined by assessing the significance of the *F*_*is*_ values by means of Fisher’s exact tests implemented in GENEPOP 4.0, with specified Markov chain parameters of 5,000 dememorization steps, followed by 1,000 batches of 5,000 iterations per batch. The sequential Bonferroni correction was applied to obtain critical confidence limits for multiple comparisons, with an initial α of 0.05. To test for significant differences in population genetic parameters between forest and grassland populations, a two-tailed *t*-test or Mann-Whitney *U* test was performed in R in accordance to assumption fulfillment.

### Genetic structure and isolation by distance

Assessment of total genetic diversity partitioned among both habitats (forest vs. grassland), among populations within one habitat, and within populations was done by carrying out a hierarchical analysis of molecular variance (AMOVA) on Euclidean pairwise distances among individuals implemented in GENALEX^73^. Individuals were assigned to two groups reflecting the two habitats. Significances were determined using permutation tests.

Parameters for pairwise genetic differentiation *F*_*ST*_ (adegenet^70^), Hedrick’s standardized measure for *G*_*ST*_ and Jost’s *D* (both calculated with the ‘mmod’ package^74^) were calculated (5000 permutations). A mantel test was used as a nonparametric equivalent of ANOVA to test the hypothesis that genetic differentiation among populations within one habitat was significantly different from differentiation among populations from different habitats^75^. Therefore we constructed a dissimilarity matrix with zeroes in the within group submatrices and ones in the between groups submatrices. If differences between habitats are greater, then the ones in the design matrix will be associated with larger differences. This ANOVA-like Mantel test was performed for the three different measures for differentiation and 5000 random permutations were used. Furthermore, a Mann-Whitney U test was performed to test for significant differences for all three differentiation parameters among habitats. The combined information of different differentiation measures enables a more profound and reliable understanding of the relations between populations^76–81^.

Evidence of isolation by distance among populations was obtained by examining correlations between matrices of genetic distances (*G*_*ST*_) and geographical distances^82^. Significance of the observed relationships was obtained by using a Mantel test^75, 83, 84^. A total of 5000 random permutations were performed. The same test was also used to look for isolation by distance separately among populations in grasslands and among populations in forests.

We also investigated whether the two habitats translated into distinct genetic clusters using a Bayesian clustering method applying Markov Chain Monte Carlo estimation implemented in STRUCTURE 2.3.4 software^48^. *K* was set to range from 1 to 10 with a Markov Chain Monte Carlo of 500 000 iterations following a burn-in of 100 000 iterations. The best *K* value was inferred from the modal value of the run with the highest log likelihood^39^. In addition, genetic clustering of populations was visualized by subjecting the *G*_*ST*_ matrix to a principal coordinates analysis (PCoA) using the *ape* package in R^85^.

### Network analysis of spatial genetic variation

We applied a network approach to spatial genetic variation known as population graphs^86^. Population graphs go beyond describing the traditional *F*-statistics and provide better insights into the overall population genetic structure by visualizing links of significant genetic similarity between populations^86, 87^ and providing quantitative measurements of link density and strength between and within habitats as estimates of population connectivity. Each node represents a population and can be connected to several other populations by edges or links. In this way, a graphic representation of the identified modules in the network can provide insights into the factors (e.g. geography, habitat differences) that define the assignment of a population to a cluster of genetically similar populations. To perform the network analysis, first a genetic distance matrix was constructed and converted into a correlation matrix in several steps following Fortuna *et al*.^87^.

Some populations are only poorly connected to others and therefore several links can be excluded in order to simplify the model without losing information about the genetic covariance structure among sites. Based on conditional independence it is possible to detect these redundant edges. Edge exclusion deviance is a theoretic measure to calculate whether an edge should be excluded from a fully saturated population graph^88, 89^. Edge exclusion was calculated as:

1 $$\tau =-N\,Ln[1-{({r}_{ij})}^{2}]$$

where *N* is the number of individuals in the entire data set, and *r*_*ij*_ is the partial correlation coefficient between sites *i* and *j*. This statistic asymptotically follows a chi-square distribution. A link among populations *i* and *j* is removed if the value of its deviance (theta) is less than 3.84 (the 5% threshold of the chi-square distribution with *df* = 1). For further details on graph theory of genetic structures see Dyer and Nason^86^ and Fortuna *et al*.^87^

### Modularity analysis

We applied a modularity analysis on the network of spatial genetic variation to detect modules or sets of populations that are more genetically related to each other than to populations belonging to other modules. Newman’s modularity^90^, *M*, can be written as:

2 $$M={\sum }_{allmodulesi}(\frac{{e}_{i}}{L}-{\sum }_{\begin{array}{c}allpairsof\\ speciesini\end{array}}\frac{{k}_{m}}{2L}\frac{{k}_{n}}{2L})={\sum }_{allmodulesi}(\frac{{e}_{i}}{L}-\frac{{d}_{i}}{2L}\frac{{d}_{i}}{2L})$$

where *e*_*i*_ is the number of edges or links within module *i*, *k*_*m*_ (*k*_*n*_) is the degree of node *m* (*n*) and *d*_*i*_ is the sum of the degrees of all nodes or patches in module *i*.

Multiple techniques consist of maximizing modularity of a complex network by exploring different possible states of the population structure. Simulated annealing is an optimization techniques that explores low cost configurations of community structure^91^ to find high level modules. In this study, we ran 100 replicates of the Guimera and Amaral algorithm^91^ for *M* and then obtained the maximum value of *M*. In order to test to what extent the value of modularity departs significantly from random expectation we ran 1000 randomizations of the network of genetic variation keeping exactly the same number of links per node, but reshuffling them randomly using a local rewiring algorithm^92^. The *P*-value was estimated as the fraction of random networks with a modularity value equal to, or higher than, the value obtained for the empirical network. The final population graph displaying the different modules was constructed using the igraph package^93^ in R 3.3.0^71^.

### Genetic inference of divergence time and historical gene flow

To estimate the timing of divergence between populations growing in grassland and forest patches, we used the coalescent-based approximate Bayesian computation (ABC) algorithm implemented in the program DIY-ABC version 2.0^94^. This software simulates one or several user specified scenarios of historic and/or demographic events and compares the observed data with summary statistics of the simulated data to calculate posterior distribution of demographic parameters^94^. Our goal was to determine the time of divergence between grassland and forest populations. Therefore, we constructed a scenario with a bifurcation event between both groups and compared it to our data. We set wide priors (10–10000) for al parameters and used 300000 simulated dataset to infer parameters. The 1% datasets with summary statistics closest to the observed data were used for approximate Bayesian computation of parameters by using regression rejection steps of the algorithm following logistic regression of the parameter values. We evaluated the goodness of fit of the model-parameter posterior distribution with the model checking function implemented in DIY-ABC version 2.0^94^. Each of the 14 summary statistics of the observed dataset is ranked against the distribution of the corresponding summary statistics from the posterior predictive distribution and deviations are checked using the estimated *P*-value.

In addition, past migration rates were estimated using MIGRATE-N 3.6^95^. MIGRATE-N uses maximum likelihood or Bayesian inference to jointly estimate historical migration rates and effective population sizes. To compensate for fluctuating population sizes at each site, we ran a simplified migration model with populations grouped per habitat type. Bayesian inference was used with one long chain of 100000 steps and a sampling increment of 1000. The default static heating scheme was used. The burn-in was set to 100000.
